# Impaired B cell anergy is not sufficient to breach tolerance to nuclear antigen in Vκ8/3H9 lupus-prone mice

**DOI:** 10.1371/journal.pone.0236664

**Published:** 2020-07-28

**Authors:** Kieran P. Manion, Yuriy Baglaenko, Nan-Hua Chang, Nafiseh Talaei, Joan E. Wither

**Affiliations:** 1 Krembil Research Institute, University Health Network, Toronto, Ontario, Canada; 2 Department of Medicine and Immunology, University of Toronto, Toronto, Ontario, Canada; 3 Division of Rheumatology, University Health Network, Toronto, Ontario, Canada; Instituto Nacional de Ciencias Medicas y Nutricion Salvador Zubiran, MEXICO

## Abstract

**Background:**

Systemic lupus erythematosus (SLE) is a severe autoimmune disease in which immune tolerance defects drive production of pathogenic anti-nuclear autoantibodies. Anergic B cells are considered a potential source of these autoantibodies due to their autoreactivity and overrepresentation in SLE patients. Studies of lupus-prone mice have shown that genetic defects mediating autoimmunity can breach B cell anergy, but how this breach occurs with regards to endogenous nuclear antigen remains unclear. We investigated whether B and T cell defects in congenic mice (c1) derived from the lupus-prone New Zealand Black strain can breach tolerance to nuclear self-antigen in the presence of knock-in genes (Vκ8/3H9; dKI) that generate a ssDNA-reactive, anergic B cell population.

**Methods:**

Flow cytometry was used to assess splenic B and T cells from 8-month-old c1 dKI mice and serum autoantibodies were measured by ELISA. dKI B cells stimulated in vitro with anti-IgM were assessed for proliferation and activation by examining CFSE decay and CD86. Cytokine-producing T cells were identified by flow cytometry following culture of dKI splenocytes with PMA and ionomycin. dKI B cells from 6-8-week-old mice were adoptively transferred into 4-month-old wild type recipients and assessed after 7 days via flow cytometry and immunofluorescence microscopy.

**Results:**

c1 dKI mice exhibited B cell proliferation indicative of impaired anergy, but had attenuated autoantibodies and germinal centres compared to wild type littermates. This attenuation appeared to stem from a decrease in PD-1^hi^ T helper cells in the dKI strains, as c1 dKI B cells were recruited to germinal centres when adoptively transferred into c1 wild type mice.

**Conclusion:**

Anergic, DNA-specific autoreactive B cells only seem to drive profound autoimmunity in the presence of concomitant defects in the T cell subsets that support high-affinity plasma cell production.

## Introduction

Systemic lupus erythematosus (SLE) is a chronic autoimmune disease in which a complex interplay of genetic and environmental factors leads to the production of pathogenic anti-nuclear antibodies (ANAs) [1]. In healthy individuals, generation of ANAs is prevented by a variety of B cell tolerance mechanisms including receptor editing, exclusion and/or deletion of self-reactive B cells prior to entry into the mature B cell compartment, induction of B cell anergy by chronic antigen engagement, lack of T cell help, and impaired differentiation into plasma cells; however, it remains unclear how defects in each of these mechanisms intersect to promote the development of lupus. The relative contribution of defects in B cell anergy to SLE pathogenesis has been of particular interest, as lupus patients show increased activation of cells with an anergic phenotype in the mature B cell compartment [2,3]; however, it is unknown whether these cells directly differentiate into antibody-secreting cells (ASCs) or instead act to inhibit endogenous antibody production by inhibiting CD4^+^ T cell activation [4] and/or inducing regulatory T (T_reg_) cells [5–7], as has been shown for some anergic B cell populations.

To address the contribution of B cell tolerance defects to the development of lupus, our laboratory has been studying congenic mice derived from the lupus-prone New Zealand Black (NZB) mouse strain [8–10]. C57BL/6 (B6) congenic mice with an NZB chromosome 1 (c1) interval extending from 170.8 to 181 Mb (c1(96–100)) possess an intrinsic B cell functional defect leading to enhanced survival, activation, and autoantibody (autoAb) production, while those with a c1 interval from 124.6–181 Mb (c1(70–100)) have additional T cell defects that drive T helper (T_h_) cell expansion and activation, resulting in fatal glomerulonephritis [11–13]. Previous work examining the c1 strains in the context of the neo-self antigen hen egg lysozyme (HEL) revealed that HEL-specific B cells from these mice produced low levels of antigen-specific IgM autoAbs, but high titres of anti-HEL and anti-ssDNA IgG autoAbs in a germinal centre (GC)-dependent fashion, indicating that anergic B cells may indeed be a viable reservoir of ASCs given sufficient T cell help. However, a major drawback of this work was that HEL is neither a cognate murine antigen nor relevant for SLE [14].

In this study, we used the V_H_3H9-Vκ8 anergy model, with heavy and light chain knock-in (KI) genes encoding a ssDNA-specific BCR (double knock-in; dKI) [15], in tandem with the c1 strains to elucidate whether the B and T cell defects in c1 mice are sufficient to breach anergy to endogenous nuclear self-antigen. We found that while c1 dKI mice retained the previously observed defects in B cell anergy and produced anti-ssDNA autoAbs, they had significantly abrogated ASCs and GCs, due in part to a lack of effective T cell help.

## Materials and methods

### Mice

B6 mice expressing KI genes for the V_H_3H9 heavy chain (IgH^a^) and Vκ8 light chain were acquired from Martin Weigert [15]. Using polymorphic marker assisted selection, KI genes (3H9 or Vκ8) were backcrossed in 3 crosses onto previously generated congenic mice with NZB c1 intervals from 96–100 cM (170.8–181 Mb) or 70–100 cM (124.6–181 Mb) [12] introgressed onto a B6 background. Heterozygous c1.3H9 mice were then crossed with heterozygous c1.Vκ8 mice to generate c1.3H9^-^Vκ8^-^, c1.3H9^-^Vκ8^+^, c1.3H9^+^Vκ8^-^ and c1.3H9^+^Vκ8^+^ animals. Genomic DNA isolated from ear notches was genotyped by PCR to identify KI-negative and dKI mice using the following primers: 3H9 F (CTGTCAGGAACTGCAGGTAAGG); 3H9 R (CATAACATAGGAATATTTACTCCTCGC); Vκ8 F (GGTACCTGTGGGGACATTGTG); and Vκ8 R (AGCACCGAACGTGAGAGG). Female mice were used for all experiments. B6, c1(96–100) and c1(70–100) animals were housed separately, while KI-negative wild type (WT) and dKI animals for each genetic background were co-housed littermates. All mice were housed in specific pathogen free microisolators with access to autoclaved food, water and environmental enrichment at the Krembil Research Institute animal facility. Animals were monitored daily by facility staff and euthanasia was performed using cervical dislocation. Experiments were conducted under protocol #123 according to the provisions of the Canadian Council on Animal Care.

### ELISAs

Serum levels of anti-ssDNA or anti-dsDNA IgM, IgM^a^, IgM^b^, IgG, IgG2a, IgG2a^a^, IgG2a^b^, and IgG2c antibodies were measured by ELISA as previously described [16]. Briefly, 96-well Immulon 2 HB plates (ThermoFisher Scientific, Rochester) were coated with ssDNA (20 μg/ml) or dsDNA (40μg/mL) derived from calf thymus DNA (Sigma Aldrich, Germany). Following blocking, plates were incubated with sera from 7.5- to 8.5-month-old female B6, c1(96–100), and c1(70–100) WT or dKI mice at a dilution of 1:100 for 1 hr at room temperature. Wells were then washed with PBS/Tween20 and incubated at room temperature for 1 hr with either alkaline phosphatase-conjugated IgM/IgG or biotinylated anti-mouse IgM^a/b^/IgG2a^a/b^. For biotinylated antibodies, plates were further incubated for 1 hr with alkaline phosphatase-conjugated streptavidin (BD Biosciences). After washing, p-nitrophenyl phosphate substrate was added (Sigma-Aldrich, Germany) and the OD (405nm) was quantified using a Wallac 1420 spectrophotometer (Perkin Elmer, Finland).

### Flow cytometry

Splenocytes were stained and analyzed as previously described [17]. Briefly, 5x10^5^ RBC-depleted splenocytes were blocked with mouse IgG (Sigma-Aldrich, Germany) for 20 min at 4°C prior to staining with directly-conjugated mAbs, including: FITC-conjugated anti-IgM^a^ (MA-69) and -CD80 (16-10A1), biotin-conjugated anti-IgM^a^ (DS-1), -CD24 (M1/69) and –Igλ51 (R11-153), and PE-conjugated anti-IgM^a^ (DS-1) and -CD138 (281–2) from BD Biosciences; FITC-conjugated anti-IgM^b^ (AF6-78) and -CD44 (IM7), allophycocyanin-conjugated anti-IFNγ (XMG1.2), -CD21 (7E9), and -CD86 (GL-1), PE-conjugated anti-IgM^a^ (DS-1), -CD23 (B3B4), -CD95 (SA367H8), and -FoxP3 (150D), PeCy7-conjugated anti-B220 (RA3-6B2) and -CXCR5 (L138D7), and Pacific Blue-conjugated CD4 (GK1.5) from BioLegend; and biotin-conjugated anti-PD-1 (J43) from eBioscience (ThermoFisher Scientific). Biotinylated peanut agglutinin (PNA) was purchased from Sigma-Aldrich (St. Louis, MI) and isotype controls were purchased from BD Biosciences. Pacific Blue-conjugated (ThermoFisher Scientific) or PerCP-conjugated streptavidin (BD Biosciences) was used to reveal biotinylated antibody staining. Live cells were detected using 0.6μg/mL propidium iodide (Sigma-Aldrich) or far-red fixable viability stain (ThermoFisher Scientific). Stained cells were acquired using a BD LSRII flow cytometer (BD Biosciences) and analyzed using FlowJo software (TreeStar, San Carlos, CA).

### Immunofluorescence microscopy

Splenic tissue was sectioned and stained as previously described [14]. Briefly, spleens were snap-frozen in OCT compound (Sakura Finetek, Torrance, CA) at the time of sacrifice. Cryostat spleen sections (5 μm) were fixed in acetone, washed with PBS, and blocked with 5% fetal bovine serum in PBS prior to staining with FITC-anti-IgD (BD Biosciences) or -anti-IgM^a^ (BD Biosciences), biotin-conjugated anti-PNA (Sigma-Aldrich), and PE-anti-IgM^a^ (BD Biosciences) or -anti-CD4 (BD Biosciences). Biotinylated antibody staining was revealed with 7-amino-4-methylcoumarin-3-acetic acid-conjugated streptavidin (Jackson ImmunoResearch) as a secondary reagent. Sections were mounted with Fluoro-Gel (Electron Microscopy Sciences) and fluorescence was visualized after 24–48 hr using a Zeiss Axioplan 2 imaging microscope (Zeiss, Oberkochen, Germany). Images were processed using ImageJ software (ImageJ, National Institutes of Health, Bethesda, Maryland).

### In vitro functional assays for B cell proliferation and activation

Splenic B cells from 2- to 10-month-old B6 or c1(96–100) dKI mice were purified using a Pan B cell negative isolation kit (Miltenyi Biotec, San Diego, CA) according to the manufacturer’s instructions. 2×10^5^ negatively-selected B cells were cultured in duplicate in media (RPMI plus 10% FBS, non-essential amino acids, L-glutamine, β-mercaptoethanol, and penicillin-streptomycin) alone or with 10 μg/mL F(ab’)_2_ anti-IgM (Jackson Immunoresearch). For CD86 expression, cells were cultured for 18 hr at 37°C, then stained with anti-B220, -IgM^a^, and -CD86 mAb (BD Biosciences) and analyzed by flow cytometry as described above. For B cell proliferation, cells were stained with CFSE, washed, and stimulated as described above, with the addition of 50ng/mL submitogenic LPS; CFSE staining was assessed after 72 hr, as previously described [12].

### Adoptive transfers

For adoptive transfers, splenic B cells were purified from 6- to 8-week-old female B6 or c1(96–100) dKI mice using a Pan B cell negative isolation kit (Miltenyi Biotec, San Diego, CA) according to the manufacturer’s instructions (post-selection purity of 90–99%). 1x10^*7*^ B cells were then stained with CFSE and injected intravenously into 4- to 5-month-old B6 or c1(96–100) WT recipients. Recipient mice were sacrificed after 7 days, and splenocytes were analyzed by flow cytometry as outlined above.

### CD4 T cell cytokine production

Splenocytes from 8-month-old mice were cultured in duplicate with media alone or with PMA (50ng/mL, Sigma-Aldrich) and ionomycin (1μg/mL) in the presence of GolgiStop (BD Biosciences) for 4 hr at 37°C. Following culture, cells were stained with anti-CD4 antibodies and then fixed and permeabilized with Cytofix/Cytoperm prior to intracellular staining for IFNγ.

### Statistics

The D’Agostino-Pearson Omnibus K2 test was used to assess normality. Mann–Whitney U non-parametric tests were used for comparisons between two groups and Kruskal-Wallis non-parametric tests with Dunn’s post test were used for comparisons between three groups. Spearman’s correlation coefficient was used to assess the significance of correlations. Asterisks indicate a p<0.05 (*), <0.01 (**), <0.001 (***) and <0.0001 (****). All statistical analyses were done using GraphPad Prism software (La Jolla, CA, USA).

## Results

### c1 congenic dKI mice show a mild breach of anergy to ssDNA

**Fig 1:**
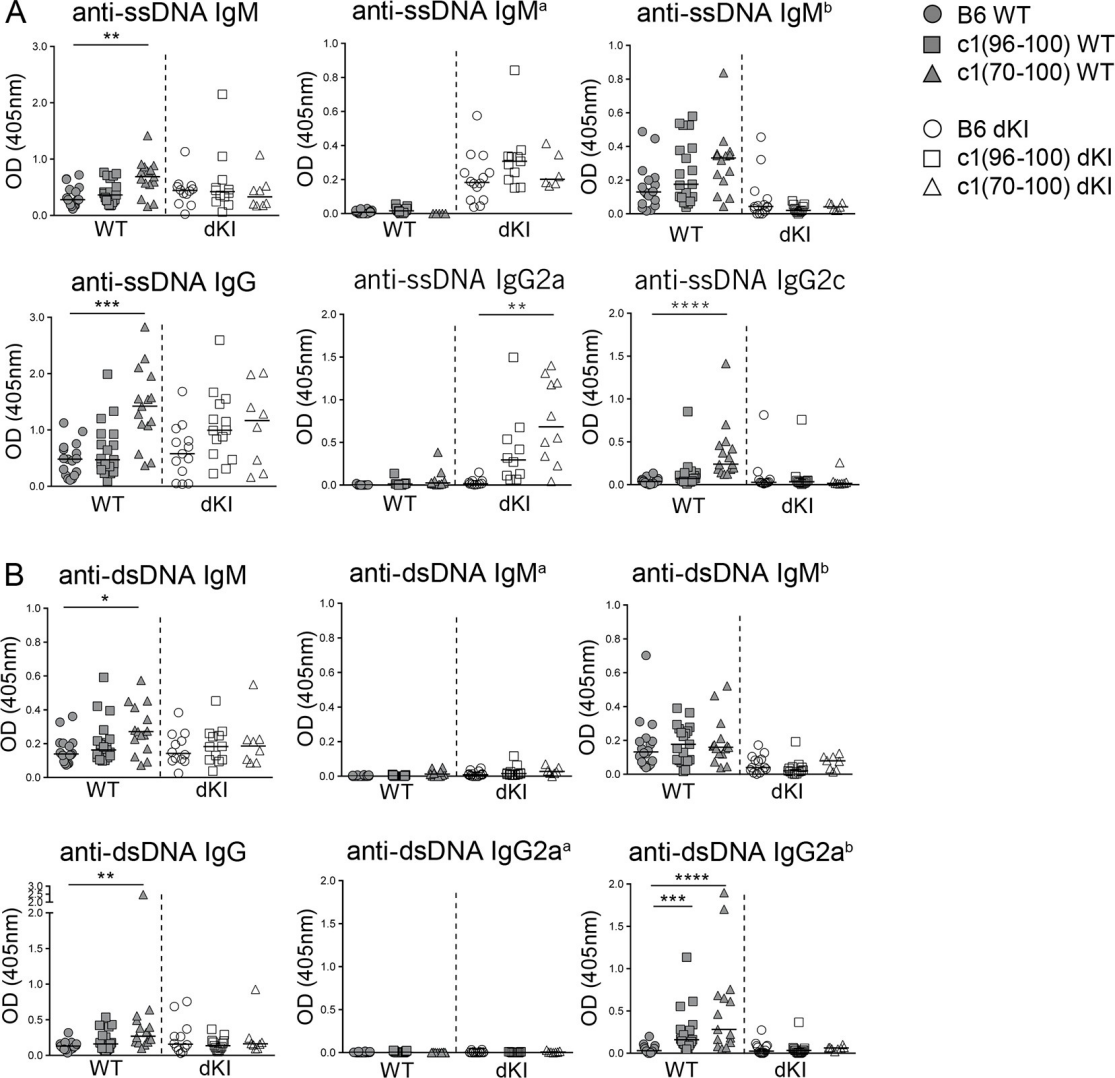
c1(70–100) dKI mice show a specific breach of B cell anergy to ssDNA. Serum from 8M B6 (circles), c1(96–100) (squares) and c1(70–100) (triangles) WT (filled) and dKI (open) mice was diluted 1:100 and assessed by ELISA for production of (A) anti-ssDNA and (B) anti-dsDNA IgM, IgM^a/b^, IgG, IgG2a/IgG2a^a^ and IgG2c/IgG2a^b^ autoAbs. Scatterplots show data from multiple independent experiments with n = 3–16 mice each. Symbols represent individual mice and lines show the median. Kruskal-Wallis non-parametric tests with Dunn’s post-test were used to compare B6 with c1 animals within each genotype. *p<0.05, **p<0.01, ***p<0.001 and ****p<0.0001.

To determine whether the altered B cell function that maps to the c1(96–100) region is sufficient to overcome anergy in nuclear antigen-reactive B cells, we crossed Vκ8 and 3H9 KI genes that encode a ssDNA-specific BCR onto the c1(96–100) background (IgH^*b*^) to produce c1(96–100) dKI mice (IgH^*a*^). As the T cell defects in c1(70–100) mice (IgH^*b*^) have been shown to augment autoAb production through a GC-dependent mechanism [14], c1(70–100) dKI mice (IgH^*a*^) were also produced to examine the role of GC tolerance mechanisms in maintaining B cell tolerance to nuclear antigens. As shown in Tables 1 and S1(gating S1 Fig), with the exception of a small decrease in the proportion of IgM^*a+*^IgM^*b+*^ cells and increase in the proportion of IgM^*a-*^IgM^*b-*^ cells, there were no significant differences in the B cell populations in c1 dKI as compared to B6 dKI mice. In all of the dKI mouse strains, > 92% of B cells expressed the IgM^*a*^ KI heavy chain paired with an Igκ light chain (Table 1). While certain light chains can mitigate the DNA reactivity of the 3H9 heavy chain, it has been shown that receptor editing is less effective in mice with a KI DNA-reactive heavy chain and that most light chain pairings with 3H9 continue to target ssDNA, suggesting that the vast majority of B cells in this model remain ssDNA-specific [18–20]. To determine whether tolerance was breached in these B cells, ANA production was assessed at 8 months of age. In line with previous findings [13,14], c1(70–100) WT mice had significantly more IgM and IgG anti-ssDNA autoAbs than B6 WT mice (Fig 1A). Although there was a trend to increased levels of IgM and IgG anti-ssDNA autoAbs in c1(96–100) WT mice, this did not achieve statistical significance as compared to B6 mice. This divergence from our previous results [14] may reflect the older age of the mice that were examined in the current study together with the increased sporadic autoAb production seen in aged non-autoimmune mice [21,22]. In dKI mice, the differences in IgM anti-ssDNA autoAb production between c1 and B6 mouse strains were lost, with low levels of IgM^*a*^ (KI-derived), but not IgM^*b*^, anti-ssDNA autoAbs being produced in all 3 stains; however, a trend to increased production of IgG anti-ssDNA autoAbs remained in c1 dKI mice. While this was not statistically significant for total IgG, the levels of IgG2a anti-ssDNA autoAbs were significantly increased in c1(70–100) dKI mice compared to B6 dKI counterparts, with intermediate levels between those in B6 and c1(70–100) observed for c1(96–100) dKI mice. As the IgG anti-ssDNA autoAbs in c1 congenic dKI mice were almost exclusively derived from the KI ‘a’ allele, these autoAbs appeared to arise from activation and differentiation of anergic dKI B cells, indicating a breach of anergy. However, the overall levels of IgG anti-ssDNA Abs in c1 dKI mice were roughly equivalent to those seen in c1 WT mice, despite a marked increase in the proportion of ssDNA-specific B cells in dKI mice, suggesting that only a very small proportion of the anergic B cells had differentiated to ASCs.

**Table 1:** Heavy and light chain usage in dKI splenic B cell subsets.

SUBSET	B6 dKI (n = 12)	c1(96–100) dKI (n = 9)	c1(70–100) dKI (n = 10)
*B220*^*+*^	52.4 [45.0, 60.9]	53.4 [47.1, 60.0]	49.6 [46.7, 59.8]
*IgM*^*a+*^*IgM*^*b-*^	84.2 [80.2, 87.6]	86.8 [85.0, 88.6]	85.6 [81.6, 91.0]
*IgM*^*a-*^*IgM*^*b+*^	1.8 [0.9, 2.9]	2.7 [1.9, 4.5]	3.6 [0.03, 5.9]
*IgM*^*a+*^*IgM*^*b+*^	10.6 [5.9, 15.1]	5.1* [1.6, 7.9]	5.0* [0.8, 7.6]
*IgM*^*a-*^*IgM*^*b-*^	2.8 [2.2, 3.6]	4.0* [3.4, 5.6]	4.8** [4.1, 6.4]
*IgM*^*a+*^*Igλ1*^*+*^	1.5 [0.8, 2.1]	1.6 [1.1, 2.2]	1.2 [0.8, 2.0]
*IgM*^*a+*^*Igλ*^*+*^	1.2 [0.9, 2.0]	1.3 [1.0, 3.7]	1.1 [0.8, 2.2]
*IgM*^*a+*^*Igκ*^*+*^	93.7 [87.7, 97.4]	92.8 [88.2, 95.0]	91.6 [81.9, 95.4]

Proportions of B220^+^ cells are expressed as a percentage of live lymphocytes. Proportions of all other cell subsets are expressed as a percentage of live B cells. Results shown are median [95% confidence interval]. Data reflects 15 independent experiments with n = 3–16 mice in each. IgM^a+^Igλ/κ proportions were measured in B6 (n = 5), c1(96–100) (n = 6) and c1(70–100) (n = 6) dKI mice. Significant differences from B6 dKI mice were determined using the Kruskal-Wallis non-parametric test and Dunn’s post-test, shown in bold; *p<0.05 and

**p<0.01.

We have previously reported that c1(70–100) WT mice produce high levels of anti-dsDNA autoAbs, which is associated with expansion of their pro-inflammatory T cell subsets [13,14]. In dKI mice, anti-dsDNA autoAbs can be produced from B cells that express endogenous IgM^*b*^ heavy chains (~2–4% of B cells, Table 1) or KI IgM^*a*^ heavy chain-expressing B cells that have acquired dsDNA specificity through light chain editing, such as those with the λ1 light chain (~1–2% of B cells, Table 1), or through somatic mutation in GCs. Surprisingly, despite the presence of T cell defects and multiple mechanisms by which anti-dsDNA autoAbs could be generated, production of anti-dsDNA autoAbs was completely abrogated in c1(70–100) dKI mice (Fig 1B).

### c1 dKI B cells demonstrate enhanced proliferation consistent with impaired anergy

**Fig 2:**
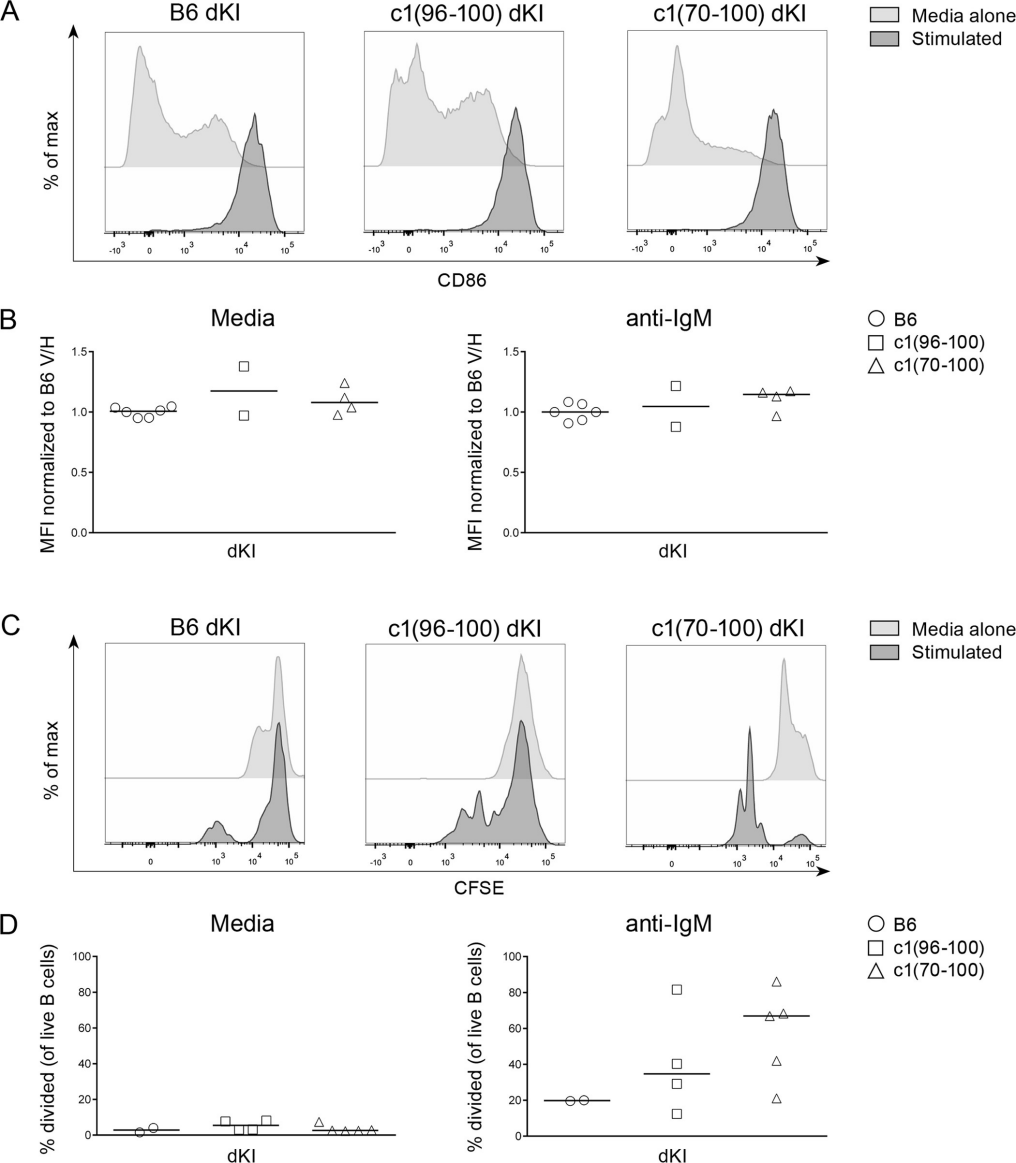
c1 dKI B cells show enhanced proliferation in vitro. (A) Histograms show the proportion of CD86^*+*^ B cells from representative B6, c1(96–100) and c1(70–100) dKI mice. Negatively-isolated splenic B cells were cultured for 18 hr in media alone (light grey) or with 10μg/mL anti-IgM (dark grey). (B) Graphs show the median fluorescence intensity (MFI) of CD86^*+*^ B cells from B6 (circles), c1(96–100) (squares) and c1(70–100) (triangles) dKI mice following 18hr culture in media alone (left) or with 10μg/mL anti-IgM (right). Values were normalized to the B6 dKI values for each experiment. (C) Histograms show proliferation of negatively-isolated, CFSE-stained splenic B cells from representative 2-10M-old B6, c1(96–100) and c1(70–100) dKI mice following 72 hr culture in media alone (light grey) or with 10μg/mL anti-IgM and 50ng/mL submitogenic LPS (dark grey). (D) Graphs show the proportion of CFSE^*+*^ B cells that have undergone at least one division from B6, c1(96–100) and c1(70–100) dKI mice. For all graphs, gates were set on the population of live B cells (PI^*-*^B220^*+*^) and data represents 4 independent experiments with n = 4–8 each. Symbols represent individual mice; horizontal lines show the median. Kruskal-Wallis non-parametric tests with Dunn’s post-test were used for statistical analysis.

Unlike other models of B cell anergy, dKI B cells do not exhibit decreased cell surface expression of IgM or altered maturation, and retain many of the functional capabilities of naïve B cells, such as the ability to mobilize calcium and upregulate CD86 following BCR crosslinking [23–25]; in agreement with this, we found that CD86 was upregulated following IgM receptor crosslinking in B6 dKI anergic B cells with no further increase seen for c1 dKI anergic B cells (Fig 2A and 2B). Additionally, while dKI B cells do not exhibit impaired survival following stimulation [25], we have previously shown that c1 B cells have a survival advantage as compared to B6 in the HEL model [14], and a similar phenomenon was observed here (S2A and S2B Fig). Instead, dKI B cells are primarily identified as anergic based on an impaired ability to proliferate in response to BCR stimulation. We previously showed that anti-HEL Ig transgenic B cells from c1(96–100) congenic mice are hyperproliferative compared with their B6 counterparts following BCR crosslinking, and that this enhanced proliferation is retained in c1 anti-HEL/sHEL double transgenic (dTg) mice, indicating impaired induction of anergy [14]. Given the paucity of B cell changes in c1 dKI as compared to B6 dKI mice, we questioned whether anergy was intact in c1 dKI B cells. To address this question, mature naïve splenic B cells were purified by negative selection from B6, c1(96–100) and c1(70–100) dKI mice, stained with CFSE, and cultured in vitro for 72 hr in media alone or with the addition of anti-IgM F(ab’)_*2*_ and sub-mitogenic LPS. Proliferation was then assessed based on the decay of the CFSE signal as measured by flow cytometry. As seen in Fig 2C and 2D, only a small proportion of B6 dKI B cells proliferated in response to stimulation as compared to media alone, confirming that most of the B cells from these mice are anergic. This proliferation was markedly enhanced for both c1(96–100) and c1(70–100) dKI B cells, indicating that a substantial subset of the B cells in these mice have impaired anergy. This is in stark contrast to the response observed in mice with a polyclonal B cell repertoire (3H9 KI only) [23], where B6 and c1 3H9 mice exhibit similar levels of B cell proliferation following stimulation (S2C Fig). Notably, the impaired anergy of c1 dKI B cells does not appear to have resulted from a lack of autoantigen (i.e. ssDNA) in c1 mice, as B6 and c1 dKI B cells had equivalently high surface levels (gMFI [95% CI]) of CD80 (B6 dKI = 6819 [6087, 7364]; c1(96–100) dKI = 4740 [2861, 9369]; c1(70–100) dKI = 5044 [2134, 8332]; p = 0.12), which has been shown to result from chronic antigen engagement in similar murine models of B cell anergy [25,26].

### c1 dKI mice have attenuated Ab-producing and GC B cells

**Fig 3:**
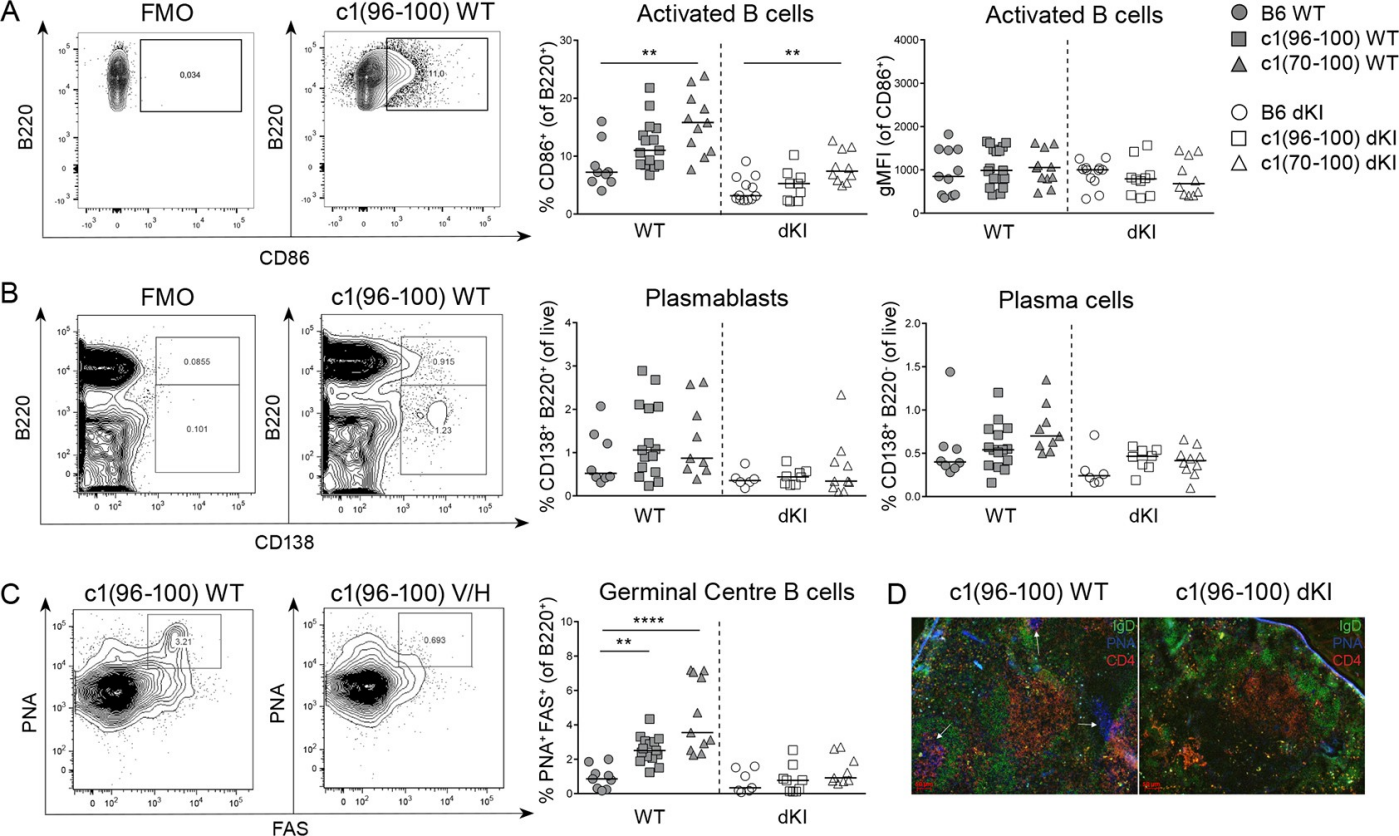
c1 dKI mice have attenuated antibody-producing and GC B cells. Splenic B cells from 8M B6 (circles), c1(96–100) (squares) and c1(70–100) (triangles) WT (filled) and dKI (open) mice were examined using flow cytometry. (A) Representative gating of activated (CD86^+^) splenic B cells (*left*) and scatterplots showing the proportions (*centre*) and gMFI (*right*) of activated splenic B cells. (B) Representative gating of plasmablasts (CD138^+^B220^+^) and plasma cells (CD138^+^B220^lo/-^) (*left*). Scatterplots show the proportions of plasmablasts (*centre*) and plasma cells (*right*) in the spleen. (C) Representative gating of GC (PNA^+^FAS^+^) B cells (*left*) and scatterplot (*right*) showing the proportion of GC B cells in the spleen. (D) Representative splenic sections from 8-month-old c1(96–100) WT and dKI mice showing the presence and absence of GCs, respectively. 6μm frozen spleen sections were fixed, permeabilized, and stained with FITC-IgD (green), PE-CD4 (red) and biotinylated-PNA revealed using SA-AMCA (blue). White arrows indicate the location of GCs. Magnification 10x. For (A)—(C), graphs show data from 15 independent experiments with n = 3–16 each. Each point represents an individual mouse and lines show the median. Kruskal-Wallis non-parametric tests with Dunn’s post-test were used to compare c1 with B6 animals within each genotype. *p<0.05, **p<0.01, and ****p<0.0001.

The lack of anti-dsDNA IgG and limited production of anti-ssDNA IgG autoAbs in c1 dKI mice, even in the presence of impaired B cell anergy, suggested that the B cells in these mice may not have received and/or responded to the signals required for ASC development. To explore these possibilities, we used flow cytometry to contrast B cell activation, recruitment into GC, and plasma cell (PC) differentiation in the B6 and c1 strains. In line with our previous research, c1(70–100) WT mice had significantly higher proportions of CD86^+^ splenic B cells than B6 WT mice (Fig 3A) with a similar trend seen in c1(96–100) WT mice, and these differences were retained in dKI mice. However, as was observed in vitro, surface levels of CD86 on activated B cells did not differ between B6 and c1 for either WT or dKI mice, indicating equivalent upregulation of CD86 in activated B cells. We also largely recapitulated prior findings of increased plasmablasts, plasma cells, and GC B cells in c1 WT as compared to B6 WT mice (Fig 3B and 3C) [14], with similar statistically significant differences observed for c1(96–100) and c1(70–100) GC B cells in the current study. The proportions of these cells were markedly reduced in all of the dKI as compared to the WT mouse strains and did not differ between B6 and c1 dKI mice. Consistent with the paucity of GC B cells in dKI mice, immunofluorescence microscopy showed a complete absence of GCs in both B6 and c1 dKI spleens (Fig 3D). Based on these results, it seems unlikely that the mild breach of tolerance seen for c1(70–100) dKI mice derives from follicular B cell activation. This is in stark contrast with our findings using the HEL B cell anergy model, where defective GC tolerance mechanisms played an important role in the observed breach of tolerance [14].

### c1 dKI mice have attenuated PD-1^hi^ T cell responses

**Fig 4:**
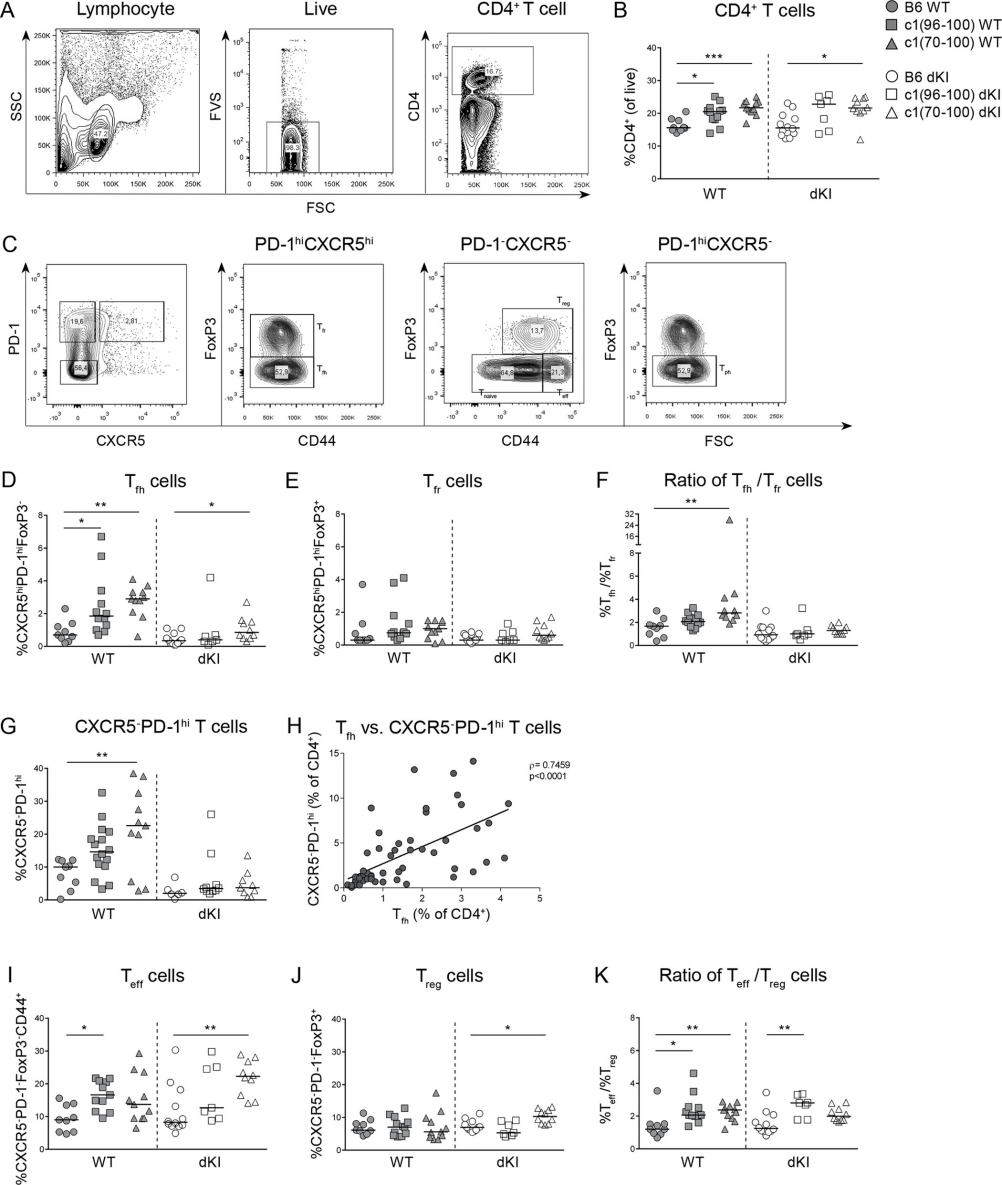
c1 dKI mice have attenuated T_fh_ and CXCR5^-^PD-1^hi^ T cells. (A) Representative plots showing the gating of live (FVS^-^) CD4^+^ T cells. (B) Scatterplot showing the proportions of live CD4^+^ T cells in B6 (circles), c1(96–100) (squares) and c1(70–100) (triangles) WT (filled) and dKI (open) mice. (C) Gating for CD4^+^ T_fr_ (CXCR5^hi^PD-1^hi^FoxP3^+^CD44^+^), T_fh_ (CXCR5^hi^PD-1^hi^FoxP3^-^CD44^+^), T_reg_ (CXCR5^-^PD-1^-^FoxP3^+^CD44^+^), T_eff_ (CXCR5^-^PD-1^-^FoxP3^-^CD44^+^) and CXCR5^-^PD-1^hi^ T cells. Graphs for (D), (E), (G), (I) and (J) show the proportions of T_fh_, T_fr_, CXCR5^-^PD-1^hi^ T, T_eff_ and T_reg_ cells, respectively. (F) and (K) show the ratio of the proportions of T_fh_ to T_fr_ and T_eff_ to T_reg_ cells, respectively. (H) Graph showing the correlation between T_fh_ and CXCR5^-^PD-1^hi^ T cells from all mice. Data represents 15 independent experiments with n = 3–16 mice each. Each point represents an individual mouse and lines show the median. Kruskal-Wallis non-parametric tests with Dunn’s post-test and Spearman’s correlation coefficient were used. *p<0.05, **p<0.01 and ***p<0.001.

We have previously shown that c1 congenic mice have elevated levels of pro-inflammatory T_h_1, T_h_17, and T follicular helper (T_fh_) cells [13]. Previous studies indicate that T_fh_ cells are essential for successful GC responses [27–29], and our laboratory has shown that this population mediates the breach of anergy to HEL in dTg c1 congenic strains [14]. Given the lack of GC B cells and plasma cells in dKI mice, we questioned whether differences in the T cell subsets that support autoAb production could explain the relative lack of autoAbs in c1 dKI mice. To this end, relevant splenic T cell populations were examined in 8-month-old B6, c1(96–100) and c1(70–100) WT and dKI mice using flow cytometry. In line with our previous work [13,14], c1 WT mice had increased proportions and numbers of CD4^+^ T cells compared to B6 mice, and this remained unchanged in the dKI strains (Figs 4B and S3A). However, while the proportion and number of T_fh_ cells were significantly higher in c1 WT than in B6 WT mice, these were largely normalized to B6 levels in c1 dKI mice with only a small but significant increase in T_fh_ cells in c1(70–100) dKI mice as compared to B6 dKI mice (Figs 4D and S3B).

T follicular regulatory (T_fr_) cells have been shown to tightly regulate GC responses by interacting with both GC B and T_fh_ cells [30,31], and there is some evidence that their development is dependent upon interactions with B cells. We therefore examined whether the proportion and number of these cells was impacted in dKI mice. Unlike T_fh_ cells, the proportion and number of T_fr_ cells did not vary significantly between B6 and c1 WT mice and similar findings were observed for dKI mice, where the proportion of T_fr_ approximated that seen in WT mice (Figs 4E and S3C). As a result, while c1(70–100) WT mice had a significantly increased ratio of T_fh_ to T_fr_ cells compared to B6 counterparts, this difference was lost for the dKI strains (Fig 4F). Taken together, the data suggest that dKI B cells lack the ability or opportunity to solicit effective T cell support for GC responses, while retaining the capacity to induce T_fr_ cells.

It has recently been discovered that a follicular-like population of T cells in the extrafollicular environment can support the differentiation of B cells into plasmablasts or long-lived plasma cells through cell-cell interactions and secretion of IL-21 [32]. These T peripheral helper (T_*ph*_) cells, which are CXCR5^*-*^ and PD-1^*hi*^, are expanded in individuals with active SLE and correlate significantly with disease scores and progression [33,34]. Emerging evidence suggests that a similar subset exists in mice [35,36], and along these lines we observed a population of CXCR5^*-*^PD-1^*hi*^ T cells that was significantly expanded in c1(70–100) WT mice compared with their B6 counterparts (Figs 4G and S3D). As occurred with T_*fh*_ cells, this difference was considerably attenuated in the dKI strains; in fact, the proportions of T_*fh*_ and CXCR5^*-*^PD-1^*hi*^ T cells correlated significantly for all strains (Fig 4H). Surprisingly, despite the loss of these extrafollicular CXCR5^*-*^PD-1^*hi*^ T cells and a decline in IFNγ-producing T cells in the c1 dKI strains (S3E Fig), there was little change in the overall proportions or numbers of global T_*eff*_ (Figs 4I and S3F) and T_*reg*_ cells (Figs 4J and S3G), or in the ratio of the two subsets (Fig 4K), between the WT and dKI mice.

**Fig 5:**
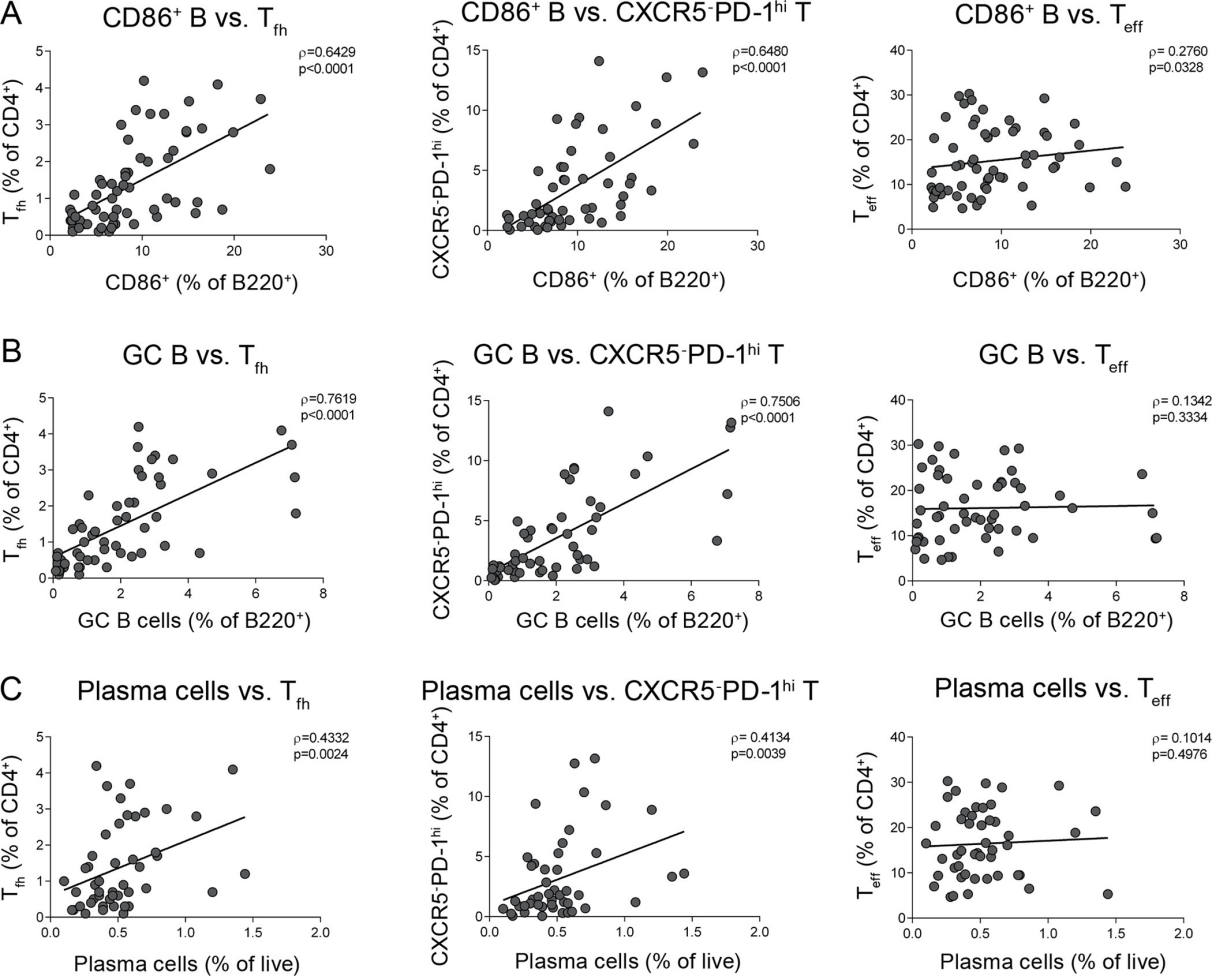
PD-1^hi^ T cells correlate with B cell activation and differentiation. Graphs showing the correlation of T_*fh*_, CXCR5^*-*^PD-1^*hi*^ T, and T_*eff*_ cells with (A) CD86^*+*^ B cells, (B) PNA^*+*^FAS^*+*^ GC B cells, and (C) CD138^*+*^B220^*lo/-*^ plasma cells for all mice. Data represents 15 independent experiments with n = 3–16 mice each. Each point represents an individual mouse. Spearman’s correlation coefficient was used to assess statistical significance.

The dramatic loss of T_*fh*_ and CXCR5^*-*^PD-1^*hi*^ T cells in dKI mice in the absence of changes to other T cell subsets suggested that the robust breach of tolerance to endogenous nuclear antigen in c1 congenic mice may be dependent upon the B cell support provided by PD-1^*hi*^ T cells in either the follicular or extrafollicular environment. In order to further investigate this possibility, we used Spearman correlations to examine the relationships between PD-1^*hi*^ T cells and the B cell subsets involved in a breach of tolerance. As can be seen in Fig 5A–5C, both T_*fh*_ and CXCR5^*-*^PD-1^*hi*^ T cells correlated significantly with the proportions of activated B cells, GC B cells and plasma cells in B6 and c1 WT and dKI mice. While B cell activation also correlated with the levels of T_*eff*_ cells, GC B and plasma cell subsets did not, reinforcing the concept that the T cell support driving the breach of tolerance in these mice is primarily derived from PD-1^*hi*^ T cells.

### c1(96–100) dKI B cells become activated and enter GCs in a c1 WT environment

**Fig 6:**
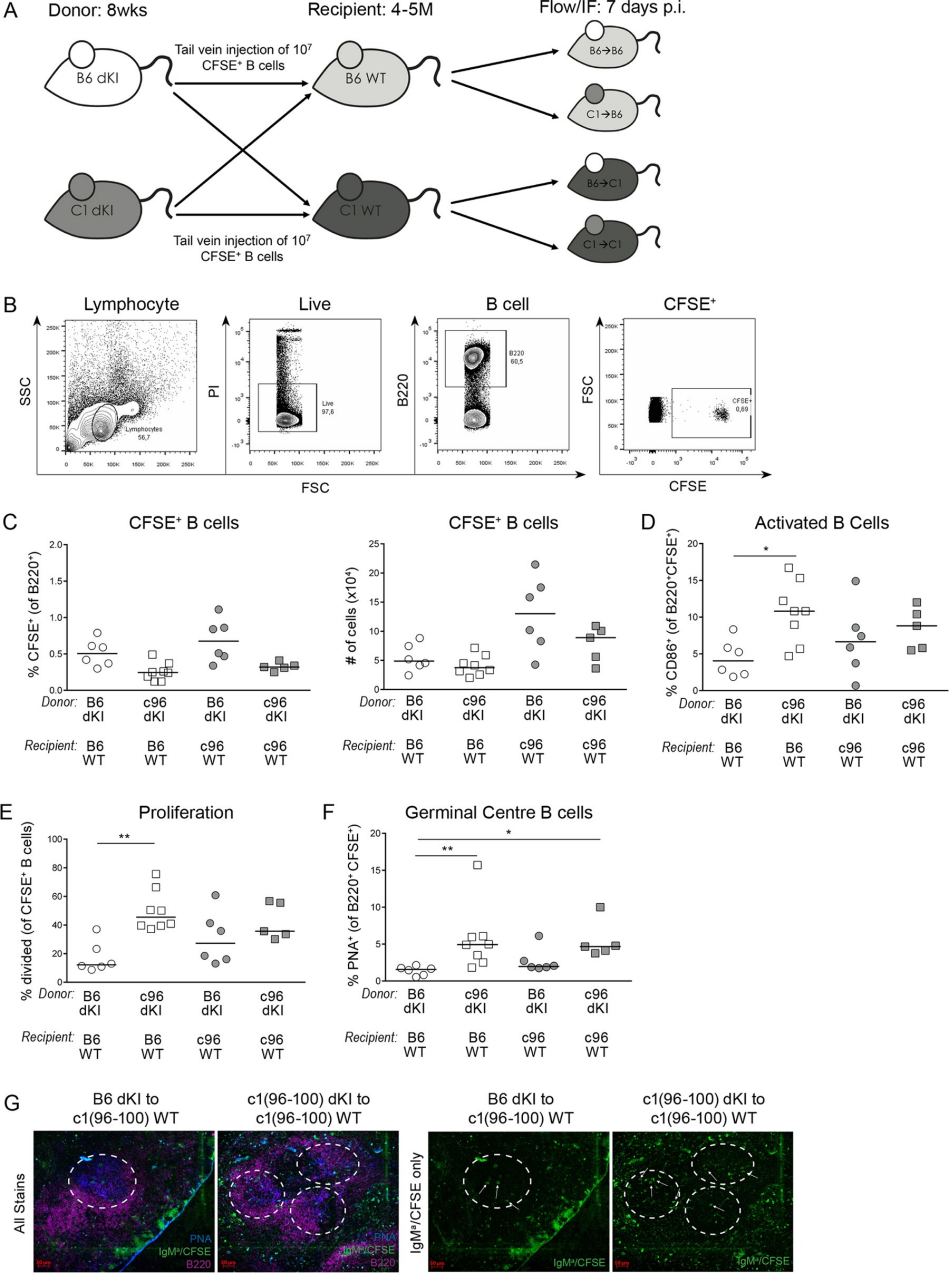
c1(96–100) dKI B cells can enter GCs in a WT environment. (A) Diagram showing the adoptive transfer model. 10^7^ splenic B cells negatively isolated from 8-wk-old B6 or c1(96–100) dKI mice were stained with CFSE and injected via tail vein into 4-5M-old B6 or c1(96–100) WT recipients. Recipient mice were sacrificed 7d post-injection and splenocytes were examined via flow cytometry and IF microscopy. (B) Representative plots showing the gating of CFSE^+^ B6 dKI splenic B cells isolated from c1(96–100) WT recipients 7d post-injection. (C) Scatterplots showing the proportions (*left*) and numbers (*right*) of transferred CFSE^+^ B6 (circles) and c1(96–100) (squares) dKI B cells in B6 (open) and c1(96–100) (filled) WT recipients. (D) Scatterplot showing the proportions of activated (CD86^+^) transferred B6 and c1(96–100) dKI B cells from B6 and c1(96–100) WT recipients. (E) Scatterplot showing the proportions of transferred CFSE^+^ B6 and c1(96–100) B cells having undergone at least one division in B6 and c1(96–100) WT recipients. (F) Scatterplot showing the proportions of GC (PNA^+^) transferred B6 and c1(96–100) dKI B cells from B6 and c1(96–100) WT recipients. (G) Representative spleen sections from c1(96–100) WT recipient mice. 6μm frozen spleen sections were fixed, permeabilized and stained with FITC-IgM^a^ (green), APC-B220 (purple) and biotinylated-PNA revealed using SA-AMCA (blue). White circles indicate the locations of GCs, while white arrows show the location of CFSE^+^ IgM^a+^ B6 dKI (*left*) or c1(96–100) dKI (*right*) B cells within the GCs. For all graphs, gates were set on the population of live B220^+^CFSE^+^ cells and data represent 4 independent experiments with n = 9–11 each. Symbols represent individual mice; horizontal lines show the median. Kruskal-Wallis non-parametric tests with Dunn’s post-test were used to determine significance. *p<0.05 and **p<0.01.

There is some evidence that anergic B cells can suppress or tolerize both T cells and other B cells [4], and that this occurs in an antigen-dependent manner. Since the dKI mouse model generates a predominantly anergic ssDNA-reactive B cell repertoire [23,24], we suspected that this preponderance of anergic cells might be preventing the breach of B cell tolerance originally observed in the c1(96–100) strain [12,14]. To test this, we created an adoptive transfer model whereby 10^7^ negatively-selected splenic B cells from 8-week-old B6 or c1(96–100) dKI mice that had been stained with CFSE were injected intravenously into 4-5-month-old B6 or c1(96–100) WT recipient mice (Fig 6A). These mice were sacrificed 7 days later and the injected CFSE^+^ dKI B cells were assessed via flow cytometry for activation, survival, and recruitment to GCs.

As seen in Fig 6B, live CFSE^*+*^ B cells were easily identifiable in recipient mice 7 days after injection. At day 7, there was no significant difference in the proportions or numbers of transferred CFSE^*+*^ B6 or c1(96–100) dKI B cells present in either B6 or c1(96–100) WT recipients, although the c1(96–100) WT environment appeared to be more amenable to B cell survival (Fig 6C). Likewise, in agreement with the results from the 8-month-old mice (Table 1), transferred B6 and c1(96–100) dKI B cells had similar proportions of cells with the IgM^*a*^ heavy chain and the Igλ1 light chain (S4A and S4B Fig). Although the proportion of CD86^*+*^ cells in c1(96–100) dKI mice was not significantly increased as compared to B6 dKI mice (Fig 3A), there was a significant increase in the proportion of CD86^*+*^ c1(96–100) as compared to B6 dKI B cells following transfer into B6 WT mice, with a similar trend observed for c1(96–100) WT recipients (Fig 6D). Furthermore, in keeping with the enhanced proliferation observed in vitro (Fig 2C and 2D), transferred c1(96–100) dKI B cells exhibited more cell proliferation than their B6 counterparts independent of the recipient animal (Fig 6E). Finally, although c1(96–100) dKI B cells were not recruited into GCs in c1(96–100) dKI mice (Fig 3C), these B cells could be recruited into GCs when transferred into c1(96–100) WT mice, and this recruitment was significantly increased as compared to that for transfer of B6 dKI B cells into B6 WT mice (Fig 6F and 6G). At day 7, very little plasmablast differentiation or autoAb production by the transferred dKI B cells was seen in any of the adoptively transferred mice, precluding examination of these phenotypes. Overall, these results reinforce our previous findings that c1(96–100) dKI B cells retain an innate capacity to breach tolerance and support the idea that their ability to do so depends largely on the surrounding environment.

## Discussion

Anergic B cells are considered a source of self-reactive ASCs in SLE as they can persist in the mature B cell repertoire and interact with T_h_ cells [37–39]; however, the exact mechanism by which anergic B cells convert to ASCs–and whether or not they are the true drivers of autoAb production–remains unclear. In this study, we found that NZB c1 lupus-prone congenic mice can breach tolerance to nuclear antigen, but that this is severely attenuated in the Vκ8/3H9 anergic model, with limited production of autoAbs despite high numbers of ssDNA-reactive cells. Furthermore, although c1 dKI B cells enter GCs when transferred into c1 WT mice, they fail to do so in the dKI environment. This appears to be related in part to a relative inability of dKI B cells to solicit the support of PD-1^hi^ T cells.

We previously showed that anergy induction is impaired in NZB c1 congenic sHEL/anti-HEL Ig dTg mice, resulting in increased proliferation and CD86 upregulation following Ig receptor crosslinking compared to B6 counterparts [14]. Here we show that this defect extends to ssDNA-reactive B cells in NZB c1 dKI mice, suggesting that the genetic polymorphism(s) in the c1 region leading to these intrinsic B cell defects act on B cells with a range of antigen affinities and specificities. It is well established that members of the *SLAM* family, particularly *Ly108*, are the primary candidate genes in the c1(96–100) interval [40,41], and there is evidence that Ly108 may alter tolerance induction through direct effects on B cell function in addition to its role in mediating GC tolerance[42–46]. Since the NZB *Ly108* allele has been shown to reduce the strength of BCR signalling and this plays an important role in anergy induction, it is probable that the NZB allelic variant leads to the impaired anergy induction and/or breach of anergy observed in these mouse strains. There may also be an additional B cell signalling defect that maps to the 70–96 interval and further disturbs B cell anergy induction in the c1(70–100) mouse strain, since proliferation of anergic dKI B cells appeared to be consistently increased in c1(70–100) as compared to c1(96–100) mice.

Although both NZB c1 congenic HEL dTg and dKI mice demonstrated a breach of anergy in vivo, there were important differences in the nature of the breach observed in the two models. In the HEL dTg model both IgM and IgG anti-HEL antibodies were produced, with the latter generated by B cells with endogenous heavy chains [14]; in contrast, dKI mice only produced IgG anti-ssDNA antibodies. More importantly, the breach of tolerance in the HEL dTg model was associated with increased numbers of GC B cells, T_fh_ cells, and plasma cells as compared to B6 dTg mice, all of which were absent in the dKI model. These findings suggest that the breach of tolerance in the HEL model is at least partially GC-dependent, while the breach in the dKI model arises through alternative mechanisms of B cell differentiation. The reasons for this dichotomy are not entirely clear but could include differences in the affinity of the Ig receptors, the abundance or localization of the antigen, or the extent/nature of T cell tolerance that may allow HEL-specific B cells to effectively interact with cognate T_fh_, whereas Vκ8/3H9 dKI B cells cannot.

Previous studies suggest that GC responses are critical for the development of pathogenic autoimmunity in lupus-prone mice and that T_fh_ cells are required for this process [14,27,30,46,47]. In keeping with our previous work [13,14], c1 WT mice had higher proportions of T_fh_ cells and GC B cells than B6 controls; however, the expansion of this cell population was abrogated in c1 dKI mice and all strains of dKI mice lacked GCs. While B cells are required for the initial differentiation of T_fh_ and T_fr_ cells [27,30], whether anergic B cells play a normal role in regulating autoreactive GC responses is unknown. In c1 dKI mice, despite evidence for impaired anergy induction, these cells appear unable to induce or sustain T_fh_ cell development, while T_fr_ cell development appears relatively unaffected. This may indicate that the co-stimulatory requirements for differentiation of T_fr_ and T_fh_ cells differ, with T_fh_ cells being more dependent on effective co-stimulation by B cells than T_fr_ cells. In this context, there is some evidence that transfer of anergic B cells into a non-anergic recipient leads to lower numbers of T_fh_ cells and it has been postulated that anergic B cells can inhibit T_fh_ cell development [4].

It is currently unclear whether the relative imbalance between T_fh_ and T_fr_ cells in c1 dKI mice plays an active role in preventing the recruitment of endogenous self-reactive B cells into GCs and their consequent differentiation into anti-ssDNA/dsDNA IgG-producing cells [48]. Although in general, autoAbs produced by endogenous B cells were absent in dKI mice, it is possible that this is due to the relatively small number of such cells (<8%) that are present, rather than active inhibition. While high levels of autoAbs were found in anti-HEL/sHEL c1 dTg mice, which had roughly equivalent proportions of cells expressing endogenous heavy chains, we cannot rule out the possibility that the increased production of ANAs in these mice as compared to the c1 dKI mice results from other features unique to the HEL model, such as the reduced life span of its B cells, which could result in increased release of nuclear antigens or elevated levels of BAFF that augment the disturbance of tolerance. Nevertheless, the possibility that the T_fh_/T_fr_ imbalance is inhibitory is consistent with recent studies suggesting that GC tolerance is regulated through the interplay of B cells, T_fh_ cells and T_fr_ cells, rather than being driven by a single cell subset. In rhesus macaques with chronic SIV infections, enhanced ratios of T_fh_ to T_fr_ cells rather than the proportion of T_fh_ or T_fr_ cells alone were found to correlate positively with the numbers of GC B cells as well as with the production of anti-dsDNA IgG autoAbs [49].

While GC responses are a primary driver of the autoAbs seen in SLE, there is also ample evidence of a role for extrafollicular T-B collaboration. The recent discovery that extrafollicular T_ph_ cells are significantly expanded in individuals with rheumatoid arthritis and SLE and are capable of promoting plasma cell differentiation has challenged the concept that robust GC responses are exclusively responsible for the production of the pathogenic autoAbs seen in autoimmune disease [32–34]. Although little is currently known about T_ph_ development or regulation in mice, the presence of a CXCR5^-^PD-1^hi^ T cell subset in c1 WT mice, and its depletion in the dKI strains, appears to suggest that, similar to T_fh_ cells, these peripheral follicular-like T cells require effective interactions with antigen-specific B cells for expansion or survival. In contrast to the inhibition of T_fh_ and T_ph_-like responses in c1 dKI mice, differentiation of conventional T_h_ and T_reg_ cells remained relatively intact as compared to c1 WT mice, with only the proportion of IFNγ-producing CD4^+^ T cells showing a reduction in c1(70–100) mice. Unlike T_fh_ cells, conventional T cells do not require B cells for their differentiation and we have previously shown that the enhanced differentiation of cytokine-producing cells in c1(70–100) WT mice arises from a combination of T cell and dendritic cell defects [13]. The findings reported herein suggest that the expansion of IFNγ-producing cells in these mice may be further augmented by self-reactive B cells and that this is attenuated when a proportion of these cells are anergic, as is the case in c1(70–100) dKI mice. Nevertheless, the presence of IgG2a anti-ssDNA antibodies in c1(70–100) dKI mice suggests that although autoreactive dKI B cells are relatively ineffective at co-stimulating T cells for IFNγ production, they can respond to previously stimulated cells, resulting in their differentiation to IgG2a ASCs.

## Conclusions

Our results show that while NZB c1(96–100) and c1(70–100) lupus-prone dKI mice have intrinsic B cell defects leading to impaired anergy induction, ssDNA-specific c1 dKI B cells demonstrate limited differentiation to ASCs and are not recruited into GCs. This appears to result from the inability of these cells to effectively induce or solicit PD-1^hi^ helper T cells, which together with a relative preservation of T_fr_ differentiation results in a skewing towards predominant suppression in the GC environment. This study reinforces the increasingly supported notion that effective T-B interactions, rather than single-cell defects, determine how and to what extent an individual breaches tolerance to self-antigen. Under this paradigm, the relative risk posed by self-reactive anergic B cells to the potential development of autoimmunity depends largely on the presence of additional, supporting defects in other immune cell populations.

## Supporting information

S1 Checklist The ARRIVE guidelines checklist. (PDF)

S1 Table Transitional, follicular and marginal zone splenic B cell subsets in WT and dKI mice. All subsets were gated on live B cells. Results shown are median [95% confidence interval]. Data reflects 15 independent experiments with n-3-16 each. Statistically significant differences from B6 WT or B6 dKI mice were measured using the Kruskal-Wallis non-parametric test with Dunn’s multiple comparisons post-test, shown in bold; *p<0.05.
(PDF)

S1 Fig Peripheral B cell subsets. (A) Representative gating of live splenic B cells (PI^-^B220^+^). (B) Representative contour plot showing the gating of IgM^a+^ and IgM^b+^ splenic B cell subsets in a B6 dKI mouse. (C) Representative contour plot showing the gating of splenic Igλ1^+^ B cells. (D) Representative contour plots showing the gating of T1 (CD24^hi^CD21^-^), T2 (CD24^hi^, CD21^int^), follicular (CD24^int^CD21^int^) and marginal zone/marginal zone-precursor (CD24^lo^CD21^hi^) B cells (*left*), and T1 (CD23^-^CD21^-^), T2-follicular (CD23^int^CD21^int^), MZ (CD23^-^CD21^hi^) and MZP (CD23^hi^CD21^hi^) B cells (*right*). All subsets were gated on the population of live B220^+^ lymphocytes.
(TIF)

S2 Fig B6 and c1 mice show similar B cell proliferation in a polyclonal B cell repertoire. (A) Histograms show the proportion of PI^-^ B cells from representative B6, c1(96–100) and c1(70–100) dKI mice. Negatively-isolated splenic B cells were cultured for 18 hr in media alone (light grey) or with 10μg/mL anti-IgM (dark grey). (B) Graphs show the proportions of PI^-^ B cells from B6 (circles), c1(96–100) (squares) and c1(70–100) (triangles) dKI mice following 18 hr culture in media alone (left) or with 10μg/mL anti-IgM (right). (C) Graphs show the proportion of CFSE^+^ B cells that have undergone at least one division from B6, c1(96–100) and c1(70–100) 3H9 mice. Gates were set on the population of live B cells (PI^-^B220^+^). Data represents 4 independent experiments with n = 4–8 each. Symbols represent individual mice; horizontal lines show the median. Kruskal-Wallis non-parametric tests with Dunn’s post-test were used for statistical analysis. *p<0.05, *p<0.01.
(TIF)

S3 Fig c1 dKI mice have decreased CD4, T_fh_, and IFNγ-producing T cells. (A)-(F) Scatterplots showing the number of live CD4^+^ splenic T cells, T_fh_ cells (CD4^+^PD-1^hi^CXCR5^hi^CD44^+^FoxP3^-^), T_fr_ cells (CD4^+^PD-1^hi^CXCR5^hi^CD44^+^FoxP3^+^), CXCR5^-^PD-1^hi^ T cells (CD4^+^PD-1^hi^CXCR5^-^CD44^+^FoxP3^-^), T_eff_ cells (CD4^+^PD-1^-^CXCR5^-^CD44^+^FoxP3^-^), and T_reg_ cells (CD4^+^PD-1^-^CXCR5^-^CD44^+^FoxP3^+^) from 8M old B6 (circles), c1(96–100) (squares) and c1(70–100) (triangles) WT (filled) and dKI (open) mice. (G) Representative gating for IFNγ-producing CD4^+^ T cells (*left*) and scatterplot showing the proportions of IFNγ-producing CD4^+^ T cells in B6, c1(96–100) and c1(70–100) WT and dKI mice (*right*). (H) Representative gating for IL-17-producing CD4^+^ T cells (*left*) and scatterplot showing the proportions of IL-17-producing CD4^+^ T cells in B6, c1(96–100) and c1(70–100) WT and dKI mice (*right*). For (A)-(F), data represents the results of 15 independent experiments with n = 3–16 each, while the data in (G)-(H) represents 8 independent experiments with n = 4–16 each. Each point represents the determination from an individual mouse and lines show the median. Statistical significance was determined using the Kruskal-Wallis non-parametric test with Dunn’s post-test. *p<0.05, **p<0.01.
(TIF)

S4 Fig Transferred dKI B cells survive in a WT environment. (A) Scatterplot showing the IgMa^+^ proportions of transferred B6 (circles) and c1(96–100) dKI (squares) B cells (CFSE^+^B220^+^PI^-^) in B6 (open) and c1(96–100) (filled) WT recipients after 7 days. IgMa^+^ gates were set on the population of live CFSE^+^ B cells. (B) Scatterplot showing the proportion of live transferred B6 and c1(96–100) dKI B cells (CFSE^+^B220^+^PI^-^) in B6 and c1(96–100) WT recipients expressing Igλ1. Data shows the results from 4 independent experiments with n = 9–11 mice each. Each point represents the determination from an individual mouse and lines show the median. Statistics were performed using the Kruskal Wallis non-parametric test with Dunn’s post-test.
(TIF)
