# Supplementary material for: Impaired B cell anergy is not sufficient to breach tolerance to nuclear antigen in Vκ8/3H9 lupus-prone mice
Source: PLoS One. 2020 Jul 28;15(7):e0236664. doi: 10.1371/journal.pone.0236664 (PMC7386585; doi:10.1371/journal.pone.0236664)
Supplement: S1 Table — All subsets were gated on live B cells. Results shown are median [95% confidence interval]. Data reflects 15 independent experiments with n-3-16 each. Statistically significant differences from B6 WT or B6 dKI mice were measured using the Kruskal-Wallis non-parametric test with Dunn’s multiple comparisons post-test, shown in bold; *p<0.05. (PDF) [file pone.0236664.s002.pdf]

**SUBSET**	**B6 WT (n=9)**	**c1(96-100) WT (n=15)**	**c1(70-100) WT (n=11)**	**B6 dKI (n=6)**	**c1(96-100) dKI (n=9)**	**c1(70-100) dKI (n=9)**
T1	6.6 [5.6, 8.0]	8.0 [6.6, 9.2]	****9.7 [7.6, 19.7]**	5.9 [3.6, 23.3]	8.8 [4.6, 19.8]	10.0 [5.0, 20.9]
T2	3.0 [1.2, 6.0]	2.2 [1.1, 4.2]	1.3 [0.1, 7.0]	2.6 [1.1, 3.9]	2.0 [1.2, 4.1]	2.2 [0.3, 3.5]
FO	72.4 [65.2, 76.2]	71.0 [62.3, 72.6]	68.4 [48.5, 70.8]	77.8 [51.6, 82.2]	74.1 [55.9, 87.1]	73.4 [53.4, 76.1]
MZ/MZP	13.7 [12.4, 21.7]	13.1 [10.2, 15.0]	13.4 [10.3, 15.8]	10.3 [8.8, 14.7]	6.7 [5.1, 12.4]	12.0 [8.7, 15.9]
MZ	8.5 [6.4, 17.7]	6.9 [4.7, 10.6]	7.9 [6.0, 9.8]	6.3 [2.7, 7.2]	4.6 [3.1, 7.8]	8.4 [6.1, 9.1]
