# Supplementary figures and images for: Impaired B cell anergy is not sufficient to breach tolerance to nuclear antigen in Vκ8/3H9 lupus-prone mice
Source: PLoS One. 2020 Jul 28;15(7):e0236664. doi: 10.1371/journal.pone.0236664 (PMC7386585; doi:10.1371/journal.pone.0236664)

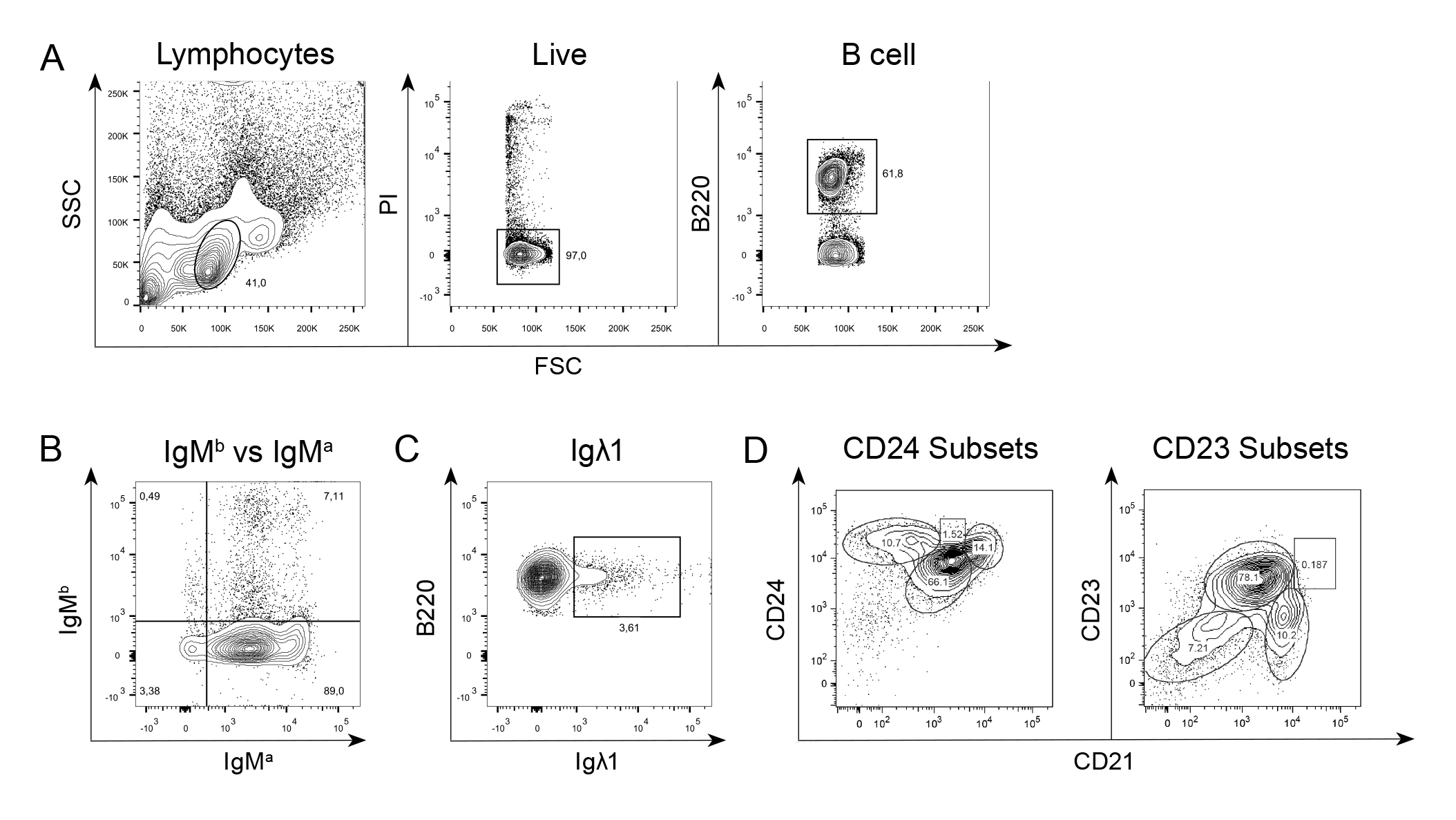

Supplement: S1 Fig — (A) Representative gating of live splenic B cells (PI-B220+). (B) Representative contour plot showing the gating of IgMa+ and IgMb+ splenic B cell subsets in a B6 dKI mouse. (C) Representative contour plot showing the gating of splenic Igλ1+ B cells. (D) Representative contour plots showing the gating of T1 (CD24hiCD21-), T2 (CD24hi, CD21int), follicular (CD24intCD21int) and marginal zone/marginal zone-precursor (CD24loCD21hi) B cells (left), and T1 (CD23-CD21-), T2-follicular (CD23intCD21int), MZ (CD23-CD21hi) and MZP (CD23hiCD21hi) B cells (right). All subsets were gated on the population of live B220+ lymphocytes. (TIF) [file pone.0236664.s003.tif]

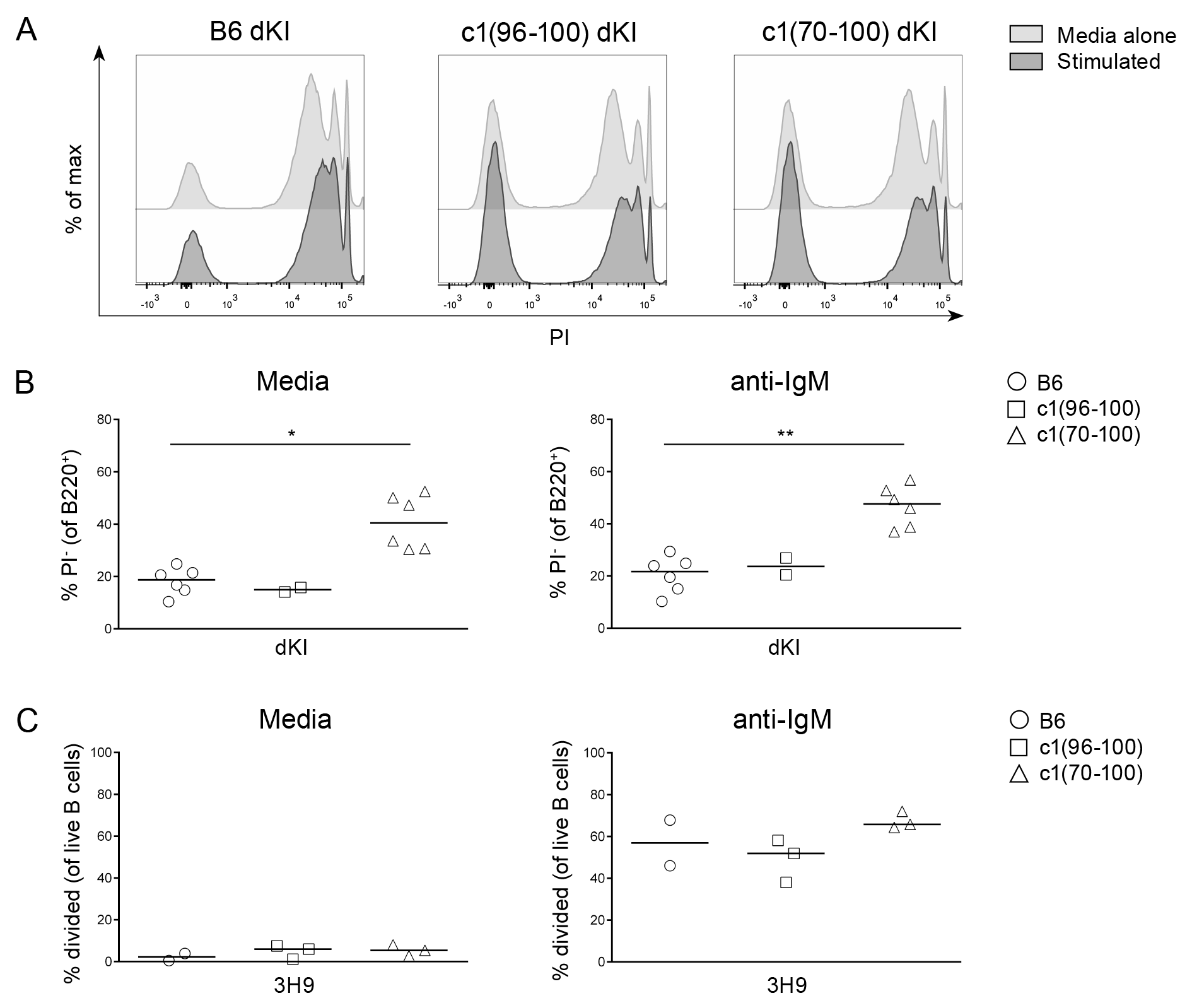

Supplement: S2 Fig — (A) Histograms show the proportion of PI- B cells from representative B6, c1(96–100) and c1(70–100) dKI mice. Negatively-isolated splenic B cells were cultured for 18 hr in media alone (light grey) or with 10μg/mL anti-IgM (dark grey). (B) Graphs show the proportions of PI- B cells from B6 (circles), c1(96–100) (squares) and c1(70–100) (triangles) dKI mice following 18 hr culture in media alone (left) or with 10μg/mL anti-IgM (right). (C) Graphs show the proportion of CFSE+ B cells that have undergone at least one division from B6, c1(96–100) and c1(70–100) 3H9 mice. Gates were set on the population of live B cells (PI-B220+). Data represents 4 independent experiments with n = 4–8 each. Symbols represent individual mice; horizontal lines show the median. Kruskal-Wallis non-parametric tests with Dunn’s post-test were used for statistical analysis. *p<0.05, *p<0.01. (TIF) [file pone.0236664.s004.tif]

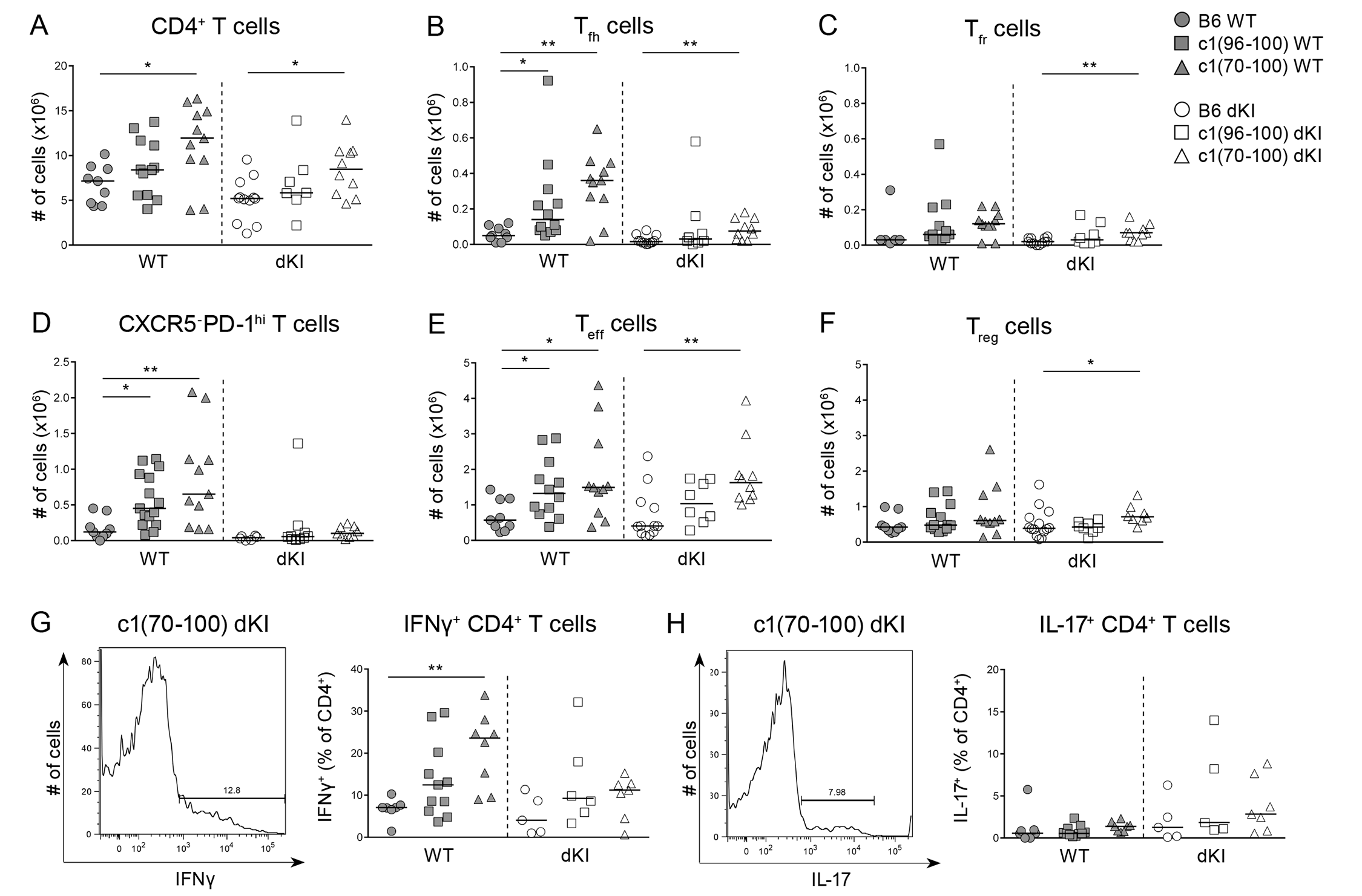

Supplement: S3 Fig — (A)-(F) Scatterplots showing the number of live CD4+ splenic T cells, Tfh cells (CD4+PD-1hiCXCR5hiCD44+FoxP3-), Tfr cells (CD4+PD-1hiCXCR5hiCD44+FoxP3+), CXCR5-PD-1hi T cells (CD4+PD-1hiCXCR5-CD44+FoxP3-), Teff cells (CD4+PD-1-CXCR5-CD44+FoxP3-), and Treg cells (CD4+PD-1-CXCR5-CD44+FoxP3+) from 8M old B6 (circles), c1(96–100) (squares) and c1(70–100) (triangles) WT (filled) and dKI (open) mice. (G) Representative gating for IFNγ-producing CD4+ T cells (left) and scatterplot showing the proportions of IFNγ-producing CD4+ T cells in B6, c1(96–100) and c1(70–100) WT and dKI mice (right). (H) Representative gating for IL-17-producing CD4+ T cells (left) and scatterplot showing the proportions of IL-17-producing CD4+ T cells in B6, c1(96–100) and c1(70–100) WT and dKI mice (right). For (A)-(F), data represents the results of 15 independent experiments with n = 3–16 each, while the data in (G)-(H) represents 8 independent experiments with n = 4–16 each. Each point represents the determination from an individual mouse and lines show the median. Statistical significance was determined using the Kruskal-Wallis non-parametric test with Dunn’s post-test. *p<0.05, **p<0.01. (TIF) [file pone.0236664.s005.tif]

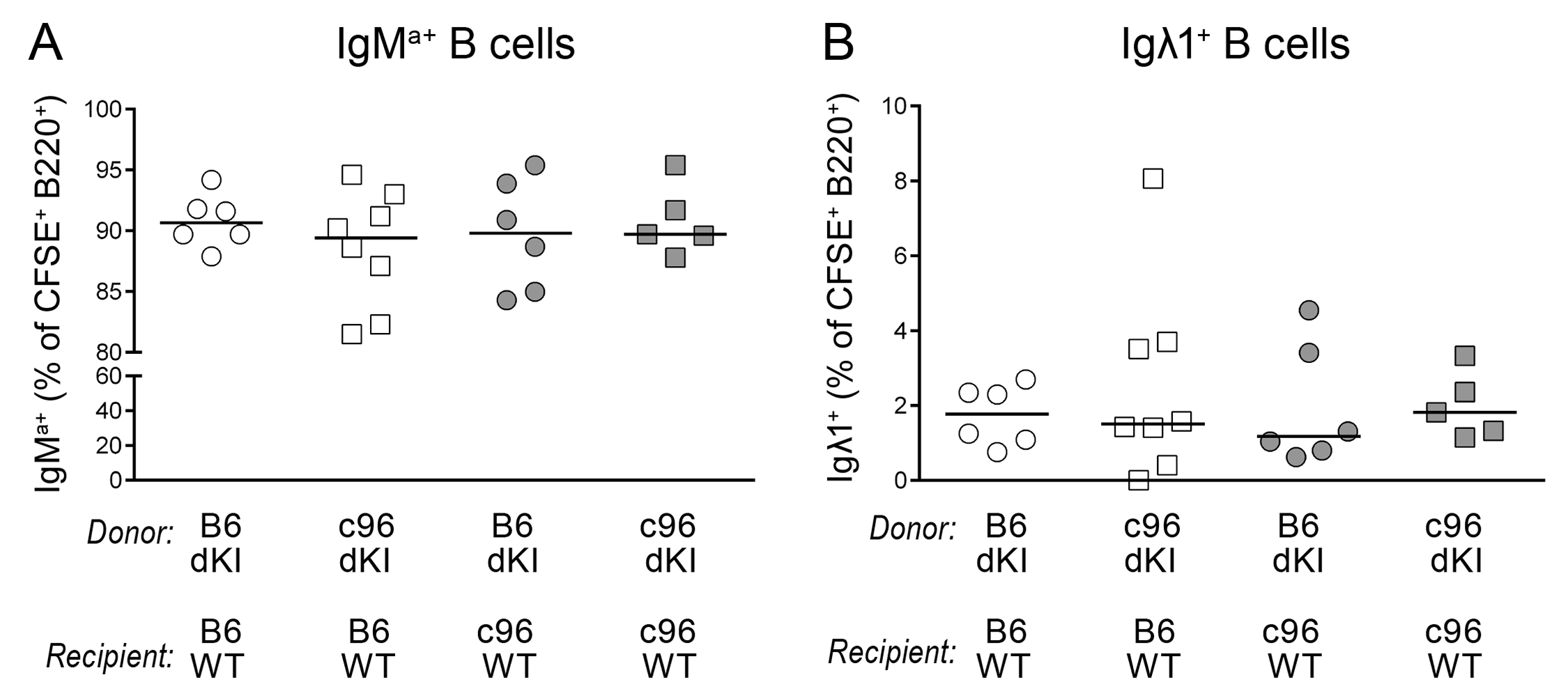

Supplement: S4 Fig — (A) Scatterplot showing the IgMa+ proportions of transferred B6 (circles) and c1(96–100) dKI (squares) B cells (CFSE+B220+PI-) in B6 (open) and c1(96–100) (filled) WT recipients after 7 days. IgMa+ gates were set on the population of live CFSE+ B cells. (B) Scatterplot showing the proportion of live transferred B6 and c1(96–100) dKI B cells (CFSE+B220+PI-) in B6 and c1(96–100) WT recipients expressing Igλ1. Data shows the results from 4 independent experiments with n = 9–11 mice each. Each point represents the determination from an individual mouse and lines show the median. Statistics were performed using the Kruskal Wallis non-parametric test with Dunn’s post-test. (TIF) [file pone.0236664.s006.tif]
